# Positive outcomes for individuals participating in psychosis research

**DOI:** 10.1177/00048674251333573

**Published:** 2025-04-16

**Authors:** Marko Milicevic, Cláudia C Gonçalves, Carl Moller, James McLure, Tracey Watson, Alison R Yung

**Affiliations:** 1 Institute for Mental and Physical Health and Clinical Translation, School of Medicine, Deakin University, Geelong, VIC, Australia; 2 School of Health Sciences, The University of Manchester, Manchester, UK

Research into psychotic disorders is essential for building evidence on the biopsychosocial causes, prevention and potential interventions which can improve lives and foster hope for individuals and their families. Findings from psychosis research in Australia help shape policies to improve clinical care. However, recruiting participants with severe mental illnesses like psychotic disorders can be challenging. Motivational deficits, a common symptom across the schizophrenia spectrum, can affect willingness to engage in research. This paper emphasizes the benefits of participating in psychosis research. We hope this will encourage people who experience or have experienced psychosis to take part in research and for clinicians to discuss clinical research opportunities with clients.

## Contribution to society

Participation in research offers individuals the chance to contribute to improving the lives of people experiencing mental health difficulties. Indeed, individuals with mental health challenges are motivated to help others facing similar difficulties and cite social responsibility and contribution to scientific progress as reasons to participate in research (Morán-Sánchez et al., 2018). Engaging in research allows individuals with psychotic disorders to be valued for their lived experience. Their insights are recognized as crucial contributions to scientific understanding, whereby experiences related to psychosis are framed as a source of unique perspective. For participants, knowing their involvement may lead to improved care and support for others can be a powerful motivator, creating a sense of agency and purpose in their mental health journey.

Unfortunately, societal stigma can lead individuals with psychotic illnesses to internalize the belief that they are unworthy or devalued in society (Corrigan et al., 2016). This is exacerbated by the high rates of people with psychosis who are not engaged with employment or education, who may feel that they lack opportunities to meaningfully contribute to their communities. By providing opportunities to participate in research that has the potential to make tangible positive changes to the care of others, some of these internalized messages can be deconstructed.

## Developing a sense of community

Research participation can bring people with similar experiences together and encourage a sense of community and belonging. This can foster empathy, understanding, connection and camaraderie, thus strengthening their sense of self-determination (McDonald and Keys, 2008). We have seen this particularly in peer-led research, where people feel open to share because they feel accepted, and their experience is valued. Our research group have adapted a group model building technique with an acute mental health population in the co-design of successful grant applications (Forrester-Bowling et al., 2025).

## Psychoeducation

### Promoting universality

In research studies, validated psychometric tools are used to assess an individual’s symptoms. Such interviewing can foster self-insight, helping participants recognize that their experiences are shared by others, which can, in turn, reduce self-stigma. Some individuals even say they are pleasantly surprised by having their symptoms, which they thought unique to themselves and ‘weird’, being actively enquired about in a structured setting. By reducing self-stigma, clinical researchers support participants in accessing more informed care and, potentially, improved outcomes.

### Symptom recognition

People experiencing distressing psychotic symptoms may be unaware their experiences are related to a mental disorder. Signs of early psychosis, such as social withdrawal, mood changes or unusual thinking, may be misattributed to other conditions such as depression, anxiety, or in the case of teenagers, described as part of the adolescent experience. Failure to recognize symptoms can significantly slow the intervention process. By advertising research studies in public spaces, particularly on social media, researchers can recruit participants from the community to provide valuable insights that help individuals contextualize their experiences and help recognize their symptoms may be linked to a mental health disorder. This approach not only aids individuals in recognizing their symptoms earlier but also helps combat stigma and can facilitate timely support, which is critical in improving long-term outcomes.

## Improving access to care

People experiencing psychotic symptoms who are not engaged with clinical services may be unaware of specialized care available to them. Research teams, often knowledgeable of local clinical care and peer support options, can play a pivotal role by providing information about the services and support available to them in their community.

Clinical research can also help address confidentiality concerns and foster open dialogue that may not always be possible in standard treatment settings. For example, ethical review processes enforce secure data handling procedures to protect participant anonymity. Detailed consent forms explain these safeguards, allowing participants to make informed decisions about their involvement. Emphasizing confidentiality and transparency can encourage individuals to openly discuss their mental health experiences. Such open discussions can help participants better prepare for potential interactions with healthcare providers, offering a model for how such discussions are typically conducted.

These benefits may be particularly pronounced during the ultra-high risk and first episode stages, where individuals are still learning about the course of their experiences with psychosis.

## Clinician referral barriers

Despite readiness to participate, clinicians treating people with schizophrenia can be hesitant to discuss research with their clients due to concerns that participation may cause them harm (Jørgensen et al., 2014). However, clinical research has many safeguards to protect participants. Research studies implement well-defined protocols, which are rigorously evaluated by ethics committees. Senior researchers and clinicians are typically centrally involved in the design and conduct of research, providing the skills and expertise necessary to manage adverse events and protect participant safety.

## Conclusion

Clinical research offers participants a meaningful opportunity to contribute to society by highlighting their unique mental health journey and experiences. The research setting provides participants with a safe and supportive framework to disclose experiences they may not yet feel comfortable sharing with friends, family or healthcare providers. Participation can encourage self-acceptance, potentially breaking down self-stigma and thus improving outcomes. Participation also provides opportunities for connection and understanding that others share similar experiences. Researchers can inform participants about treatment options available to them. We encourage mental health clinicians to discuss clinical research opportunities with their clients and discuss the aforementioned benefits to help overcome motivational barriers. In addition, clinicians should ask clients for their thoughts on participation, allowing them to make informed decisions on whether they would like to participate, thus fostering their sense of self-determination. Researchers can inspire potential participants by conveying these benefits via social media and other community connections such as youth centres and health clinics. These suggestions will contribute to improving engagement in psychosis research, thus helping to advance mental health policies and evidence-based treatments for people living with psychosis.
